# Regulation of Growth and Metabolism by Amino Acid–Modified Diets in Animals: Implications for Animal Husbandry

**DOI:** 10.1111/asj.70261

**Published:** 2026-11-13

**Authors:** Hiroki Nishi, Suguru Fujita, Fumihiko Hakuno

**Affiliations:** ^1^ Department of Science and Engineering Saitama University Saitama Japan; ^2^ Department of Animal Resource Sciences, Graduate School of Agricultural and Life Sciences The University of Tokyo Tokyo Japan

**Keywords:** amino acids, growth control, lipid metabolism, protein nutrition

## Abstract

Protein is an essential nutrient for growth, and it has long been established that feeding a low‐protein diet (LPD) leads to growth retardation due to insufficient intake of essential amino acids. However, accumulating evidence has revealed that LPDs can extend both healthspan and lifespan across a variety of organisms. Furthermore, multiple studies have shown that LPDs influence not only growth but also a wide range of physiological functions, including systemic glucose and lipid metabolism, cognitive performance, and immune responses. Notably, many of these phenotypes can be independently induced by restricting or supplementing specific amino acids in the diet. The underlying mechanisms are being extensively investigated across fields ranging from basic molecular biology to applied animal sciences. Traditionally, protein nutrition in livestock has focused primarily on optimizing growth rates and improving yield; however, emerging evidence suggests that dietary amino acid manipulation may also enable value control of meat products such as fatty liver (foie gras) and marbled meat, potentially redefining the role of protein nutrition in animal farming. This review summarizes how the quantity and quality of dietary protein affect growth and metabolism in animals and explores how findings from model organisms can be translated into livestock science.

## 1 Introduction

Protein, carbohydrate, and fat are called macronutrients, which are essential for animal survival, reproduction, and homeostasis. All of them can be used in the animal body as biological fuels, and thus, every heterotroph relies exclusively on these nutrients for their daily energy demand. Unlike carbohydrates and lipids, which are generally the primary energy source or reservoir, proteins are unique in that they are a sole nitrogen source and make a greater contribution to constructing the animals' body in both physical and molecular aspects than to energy production. Hence, prolonged insufficient protein intake from diet is often associated with undesirable physiological outcomes such as growth retardation and muscle atrophy most representatively (Kim et al. 2025). Amino acids, the monomeric constituents of proteins, are traditionally categorized into either essential (indispensable) or nonessential (dispensable) amino acids. Essential amino acids (EAAs), including lysine, histidine, methionine, threonine, leucine, isoleucine, valine, phenylalanine, and tryptophan, are not synthesized de novo in humans, rodents, and many other animal species, and thus must be obtained through the diet. In contrast, nonessential amino acids (NEAAs) can be endogenously synthesized, whereas a part of them such as arginine, tyrosine, and cysteine are produced insufficiently to meet biological demand in some circumstances, which are sometimes called conditionally EAAs also. Historically, animal nutritional science has primarily focused on establishing the quantitative requirements of EAAs, particularly in the context of animal growth and productivity, because NEAAs have been regarded as “dispensable.” The concept of protein “quality” has therefore often been interpreted as the ability of a dietary protein source to satisfy the EAA requirements (e.g., amino acid score) (Report of an FAO Expert Consultation 2013).

However, recent research has expanded this conventional view. Numerous studies have shown that protein restriction—independent of caloric restriction—elicits a diverse range of physiological adaptations, including modified glucose and lipid metabolism, enhanced insulin sensitivity, and even lifespan extension in multiple species including yeasts, fruit flies, fish, poultry, pigs, rodents, and even humans (Bruce et al. 2013; Hill et al. 2022; H. Hu et al. 2024; Kitada et al. 2019; Levine et al. 2014; McCay et al. 1928; Mirzaei et al. 2016; Solon‐Biet et al. 2014; C. Zhu et al. 2022). Beneficial effects of protein restriction have also been observed in disease models such as Alzheimer's disease model mice and tumor‐bearing mice (Babygirija et al. 2024; Mulkeen et al. 2025), suggesting that it positively affects the immune and cognitive functions. Our research group has also contributed to this field by systematically evaluating the effects of whole protein‐ and single amino acid‐restricted diets on animal physiology and metabolism using rodent models: We have shown that protein restriction not only impairs growth but also induces ectopic fat deposition in the liver (Nishi et al. 2018, 2022), and skeletal muscle (Goda et al. 2021), increased insulin sensitivity (Toyoshima et al. 2010), decreased gluconeogenesis (Toyoshima et al. 2010), and hypertrophy of subcutaneous adipose tissue (Goda et al. 2021). Remarkably, many of these effects can be induced by diets in which only specific amino acids are restricted: Arginine restriction promotes fat accumulation in the liver (Otani et al. 2020), lysine restriction leads to intramuscular fat deposition (Goda et al. 2021), BCAAs restriction impairs insulin secretion from pancreatic beta cells (Horiuchi et al. 2017), methionine or serine restriction compromises T cell expansion (E. H. Ma et al. 2017; Roy et al. 2020), valine restriction disrupts proper maintenance of hematopoietic stem cells in the bone marrow (Taya et al. 2016), and so on. In more basic research, some amino acids are proved to bind directly to their corresponding effectors either allosterically or covalently, thereby resulting in the subsequent metabolic regulations through downstream transcription factors and enzymes (He et al. 2017; X. Hu and Guo 2021). These findings provide strong evidence that amino acids act not only as constituent pieces or fuels of the body but also as signaling molecules that regulate metabolic homeostasis in a tissue/organ‐specific manner. Also, it is worth mentioning that these diverse bioactivities are exerted irrelevantly to whether the amino acid belongs to EAA or NEAA.

In this review, we aim to provide a comprehensive synthesis of recent findings regarding how dietary amino acid composition associates growth, metabolism, and tissue‐specific physiology. Besides, we will discuss the potential applications of these mechanistic understandings to livestock science, sustainable animal production, and future food innovation.

## 2 Dietary Amino Acid Modulation and Its Impact on Growth Regulation in Animals: Significance of GH/IGF‐I Axis

The study of protein nutrition began to bud in the early nineteenth century, when a French physiologist, François Magendie, and his colleagues carried out a series of experiments, which demonstrated that a nitrogenous component in a diet was essential for animals to live and grow (Li 2025; Raubenheimer and Simpson 2016). Later, a German chemist, Justus von Liebig, introduced the classification of macronutrients—carbohydrate, fat, and protein—and established the foundation of protein nutritional science (Li 2025; Stafford 2010). By the mid‐twentieth century, chemists and biochemists such as Lafayette B. Mendel and Thomas B. Osborne conducted controlled feeding experiments using rats showing that the absence of specific amino acids could halt growth, and William C. Rose identified threonine as the last of the 20 proteinogenic amino acids, successfully conceptualizing EAAs in both rodents and humans (Simoni et al. 2002a, 2002b). From these on, protein quality, as well as quantity, has attracted great interest from researchers working on animal nutrition and growth.

In a general assumption, feeding an LPD is equivalent to a deficiency of EAA intake, which results in growth impairment. Because a shortage of EAAs has been regarded as a shortage of the building blocks of body proteins, that is thought to be an immediate reason for the growth retardation; however, the actual mechanism involved is rather more complex. One of the most important endocrine systems involved in the growth regulation is growth hormone (GH)/insulin‐like growth factor (IGF‐I) axis (Figure 1). Briefly, GH is released, in an oscillatory manner, from the anterior pituitary gland in response to the stimulation of GH‐releasing hormone (GHRH) and acts on the liver, where it enhances *Igf1* mRNA transcription through JAK2/STAT5b pathway (Giustina and Veldhuis 1998; Rotwein 2012). Although IGF‐I is synthesized in many tissues, majority of the circulating IGF‐I is derived from the liver (Yakar et al. 1999). When the IGF‐I concentration in the blood exceeds a threshold, it in turn inhibits GHRH activity, making a feedback loop. IGF‐I in the blood is usually found in a form of ternary complex with acid‐labile subunit (ALS) and IGF‐binding proteins (IGFBPs) (LeRoith et al. 2021). Under normal conditions, most IGF‐I is present in complex with IGFBP3, which significantly extends the half‐life of IGF‐I in the bloodstream, whereas binding to IGFBP1 restricts IGF‐I bioactivity and it is rapidly cleared out of the blood circulation (LeRoith et al. 2021).

**FIGURE 1:**
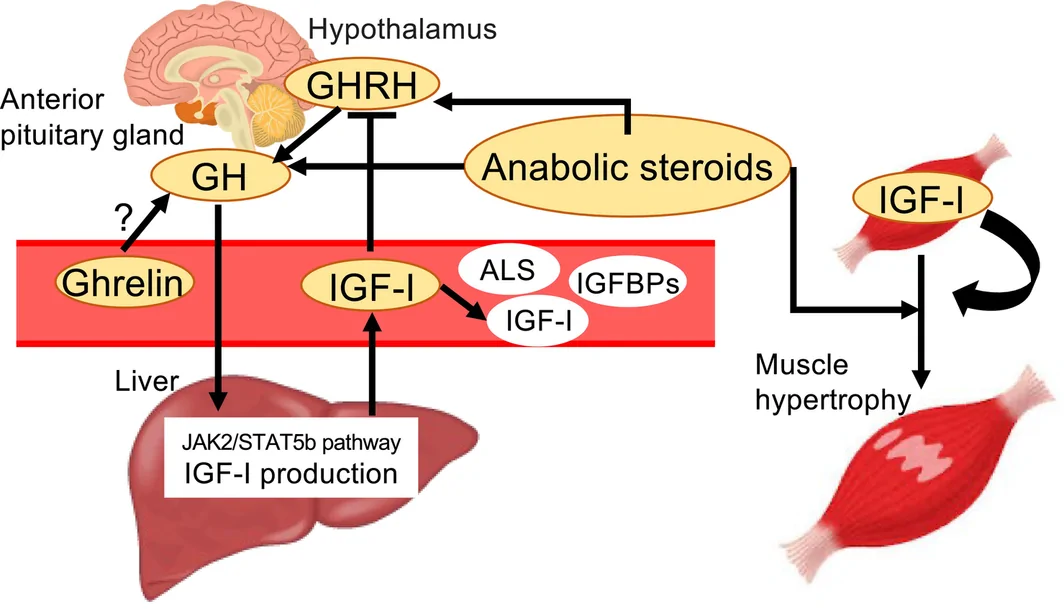
GH is released from the anterior pituitary gland in response to GHRH. GH stimulates IGF‐I production in the liver through JAK2/STAT5b pathway. IGF‐I in the blood usually forms a ternary complex with ALS and IGFBPs. Peripheral tissues such as skeletal muscle also express IGF‐I locally in a GH‐independent manner, which is likely to contribute to increasing muscle. Anabolic steroids promote muscle hypertrophy through both modulating GH/IGF‐I axis and acting directly on muscle metabolism. ALS, acid‐labile subunit; GH, growth hormone; GHRH, GH‐releasing hormone; IGF‐I, insulin‐like growth factor‐I; IGFBP, IGF binding protein; JAK2, janus kinase 2; STAT5b, signal transducer and activator of transcription.

Several studies have indicated that nutritional status or dietary protein has much influence on the GH/IGF‐I axis at multiple steps. In a rat model, feeding an LPD reduces the magnitude, but not frequency, of GH secretion; decreases circulating IGF‐I levels; and enhances IGFBP1 production in the liver, all of which lead to inhibition of IGF‐I anabolic activity (Harel et al. 1995; Oster et al. 1995; Takenaka et al. 1993; Takenaka, Oki, et al. 2000). A similar reduction in circulating IGF‐I in response to low protein intake has also been observed in middle‐aged humans, whereas this association was not observed in adults aged > 65 years (Levine et al. 2014). Consistently, in cultured hepatocytes, amino acid deprivation in the medium blunts the ligand‐dependent activation of GH signaling and IGF‐I transcription and increases IGFBP1 mRNA levels (Saito et al. 2024; Takenaka, Komori, et al. 2000). Ghrelin, so called a hunger hormone, is another GH secretagogue, which responds to protein feeding (Khatib et al. 2014). In contrast to the general belief that plasma ghrelin level rises before a meal and drops shortly after a meal, some researchers argue that such an effect of feeding on ghrelin secretion attributes to dietary carbohydrates, and they have shown that high‐protein diet or oral EAA administration even enhances ghrelin secretion (Erdmann et al. 2003, 2004; Knerr et al. 2003). That implies that protein‐feeding‐stimulated ghrelin secretion can potentiate GH activity, which may underlie the dietary protein/EAA‐dependent growth promotion. However, contradicting results also exist as to ghrelin that protein‐rich diet decreased the plasma ghrelin concentrations to a greater extent than carbohydrate‐rich diet (Blom et al. 2006). In these conflicting studies, the experimental settings are comparable; the methods of sampling and measurement are similar. Differences in protein source, amino acid composition, and the carbohydrate and fat contents of the test meals may partly contribute to the conflicting ghrelin responses: However, the available evidence does not identify a single factor that explains the discrepancy. Moreover, most studies have examined acute postprandial responses, and evidence regarding the effects of chronic low‐protein feeding on ghrelin secretion remains limited. Thus, the contribution of ghrelin to dietary‐protein‐responsive metabolic regulation still remains controversial. These observations suggest that amino acid deficiency negatively affect the entire activity of GH/IGF‐I axis, which may be one of causes of growth retardation under protein‐restricted conditions. In addition, several reports have shown that both endogenous and exogenous GH failed to increase blood IGF‐I levels and to promote growth in protein malnourished rats, steers, and humans (Ammann et al. 2010; Breier et al. 1988a; Fazeli and Klibanski 2014; Isley et al. 1983; Soliman et al. 1986; J. P. Thissen et al. 1990; J.‐P. Thissen, Triest, et al. 1991), whereas conflicting results have also been reported as to the growth enhancement by IGF‐I administration (Nishi et al. 2022; J.‐P. Thissen, Underwood, et al. 1991). In cattle and poultry, LPD feeding rather increases the blood GH concentrations in contrast to rodents, whereas IGF‐I levels are significantly downregulated (Breier et al. 1988a; Lauterio and Scanes 1987). These facts indicate that protein restriction can cause GH resistance simultaneously, and the IGF‐I activity, rather than GH levels, seems more relevant to LPD‐induced growth defects.

In a meat industry, hormonal growth promotants are often used to make livestock animals grow fast and to improve feeding efficiency. GH, androgen (trenbolone acetate), and estrogen (17β‐estradiol) are often used as growth promoters, and its anabolic effects have been demonstrated in many animal species including rats, pigs, cattle, and humans (Evans et al. 2022; Fernández‐Pérez et al. 2013; Grant et al. 1991; Leung et al. 2004). Although steroid hormones are more commonly used for practical reasons, many of anabolic activities exerted by them are mediated via the GH/IGF‐I action, confirming the importance of GH/IGF‐I axis in the meat production (Figure 1) (Carson and Manolagas 2015; Gharahdaghi et al. 2021). In particular, close relationship between GH secretion/action and estrogen concentrations, sex, puberty, or estrous cycle is well documented as to rodents, cattle, and humans (Evans et al. 2022; Fernández‐Pérez et al. 2013; Leung et al. 2004). However, the effects of steroid administration and subsequent GH upregulation on IGF‐I expression are variable among tested conditions, route of administration, and species; some reported that estrogen implant enhanced the IGF‐I production in the liver of cattle, whereas another report showed that estrogen administration decreased the serum IGF‐I levels in human (Evans et al. 2022; W Kam et al. 2000). Furthermore, exogenous estrogen administration can decrease IGFBP3 levels and increase IGFBP1 production in human, both associated with mitigation of IGF‐I activities (Heald et al. 2000; W Kam et al. 2000). Remarkably, the (muscle) growth promoting effect of anabolic steroids can be enhanced synergistically by dietary protein intake and abolished by food restriction or dietary arginine deficiency (Albaz et al. 2023; Breier et al. 1988a, 1988b; Cremades et al. 2004). Given all these facts above, anabolic activities of the growth promotants are mediated, at least in part, through GH/IGF‐I axis, and importantly, its effectiveness can vary in a context‐dependent manner. Considering that dietary proteins are the direct modulator of systemic IGF‐I action, the nutritional status of animals―especially protein―is one of the most notable factors that determines the efficacy of growth promotants.

It must be mentioned that liver‐specific IGF‐I depletion leads to a marked reduction in circulating IGF‐I levels by ~75% in mice, yet they show no apparent growth defects, although systemic IGF‐I knockout mice exhibit lethal defects in growth and development (Powell‐Braxton et al. 1993; Yakar et al. 1999), which questions the contribution of liver‐derived IGF‐I circulating in the blood to the animal growth regulation. Instead, evidence have illustrated that IGF‐I produced locally in extrahepatic tissues is worthy of notice; exogenously administered GH and steroid hormones increased IGF‐I expression in skeletal muscles apart from the involvement of the liver, and skeletal muscle‐specific overexpression of IGF‐I causes muscle hypertrophy without affecting serum IGF‐I concentrations (Musaro et al. 2001; Pampusch et al. 2008; Smith and Johnson 2020; Velloso 2008; Zou et al. 2018). Skeletal muscle‐specific ablation of local IGF‐I production leads to a decrease in muscle mass with little influence on bone development (Barton et al. 2012; Bikle et al. 2015; Vassilakos et al. 2019; Yoshida and Delafontaine 2020). Furthermore, local expression of IGF‐I in the diaphragm muscle is reportedly downregulated under the malnourished condition in rats (Lewis et al. 2003). Collectively, it is likely that local IGF‐I, which acts in an autocrine/paracrine manner, rather than GH/IGF‐I in the systemic blood circulation, may be more immediately significant in lean mass gain and its efficiency is strikingly influenced by the nutritional environment of the animals (Figure 1).

## 3 Dietary Amino Acid Modulation and Its Impact on Metabolic Regulation in Animals

Dietary amino acids, traditionally viewed as building blocks for protein synthesis, are now recognized as potent modulators of systemic metabolism, growth, and health in both model organisms and domesticated animals. Recent research has demonstrated that manipulating dietary amino acid composition—without altering caloric intake—can elicit profound effects on glucose and lipid homeostasis, immune function, gut health, and lifespan. Because many of these studies are conducted aiming virtually for clinical nutrition, “health benefits” of dietary modification including preventing from diseases, improving physiological dysfunctions, and extending heathspan have more likely been focused on. Contrastingly, in management of livestock animals and meat production, “benefits” are highly context‐dependent, sometimes even opposite to clinical benefits. For example, some of the “disease” states such as gigantism, muscle hypertrophy, hypogonadism, hepatosteatosis, and myosteatosis are often considered as preferable traits in a certain case. Therefore, understanding the implications of dietary amino acids for both health and disease will provide broader insights in animal production systems, offering opportunities to reconsider the feeding strategies that enhance product quality, maximize feed‐to‐gain ratio, and align with sustainable management of livestock and environment.

### 3.1 Amino Acid Restricted Diets

Several studies have clearly demonstrated that moderate protein restriction in a certain stage of animal raising will modulate or improve carcass traits and meat quality. Mild reduction of dietary protein can thicken backfat and abdominal fat; increase intramuscular fat levels (marbling), tenderness, juiciness, and meaty flavor; and improve feed conversion ratio and nitrogen efficiency in pigs and broiler chickens (Allameh and Toghyani 2019; Hilliar et al. 2020; Teye et al. 2006; D. Wang et al. 2025; J. Wang et al. 2012). Along with the growing knowledge on the physiological impact of LPDs, accumulating evidence have illustrated that single amino acid–restricted diets also show diverse effects on animal physiology, which are seemingly different from those of LPDs. Of those, methionine restriction (MetR) and branched‐chain amino acids (BCAA: valine, leucine, and isoleucine) restriction (BCAAR) has been most extensively studied for its multiple effects on metabolic health and longevity. Dozens of studies have revealed that MetR, without reducing caloric intake, can delay growth, decrease lean mass and adiposity, improve insulin sensitivity, and mitigate age‐related disorders, resulting in extension of healthspan and lifespan in yeasts, flies, rodents, and potentially humans (Brown‐Borg and Buffenstein 2017; Mcisaac et al. 2016; Miller et al. 2005; Parkhitko et al. 2021). In addition, MetR coupled with choline deficiency is the established model diet for induction of severe metabolic dysfunction‐associated fatty liver disease (MAFLD) (Stephenson et al. 2017). On the other hand, several reports have demonstrated that dietary BCAA consumption and the blood BCAA concentration correlate with the incidence of obesity, insulin resistance, and type 2 diabetes, and BCAAR can be beneficial to restore these phenotypes, in both rodents and humans (Cummings et al. 2018; Fontana et al. 2016; Zheng et al. 2016). Although both restriction models exhibit slower body weight gain, because methionine and BCAAs are EAAs, they substantially affect body composition of the animals including adiposity, and lean: fat ratio.

Mechanistically, a number of researchers have associated restriction of these amino acids with modified oxidative status, altered one‐carbon metabolism, decreased IGF‐I activity, and increased fibroblast growth factor 21 (FGF21) signaling (Figure 2) (Babygirija and Lamming 2021; Ling et al. 2023). Methionine is the primary sulfur source of many sulfur‐containing metabolites in animals, including cysteine, taurine, glutathione, and S‐adenosylmethionine (SAM), and particularly, SAM is known as a main methyl donor in cells, which is used by a series of methyltransferases to methylate metabolites, nucleic acids, proteins, and even organelles (Mentch and Locasale 2015). Methionine and choline deficiency perturbs the cellular one‐carbon metabolism, causing SAM shortage, which in turn modifies the epigenetic profile in each tissue. Because many studies have suggested the remarkable link between epigenetic changes and MAFLD and GH/IGF‐I axis, a part of MetR‐induced metabolic changes have been explained through this mechanism (Álvarez‐Nava and Lanes 2017; Lee et al. 2014). Furthermore, methionine (or SAM) and leucine are the established activators of mechanistic target of rapamycin complex (mTORC1), which signals to the downstream effectors and is involved in diverse metabolic regulations (X. Hu and Guo 2021). In addition, GCN2 kinase, another amino acid‐responsive factor, is also activated by MetR and BCAAR, and its activation is well known to enhance FGF21 production in the liver, which again is involved in diverse metabolic regulations (X. Hu and Guo 2021). Although a concrete molecular link between dietary MetR or BCAAR and metabolic outcomes has not been fully understood to date, these factors apparently contribute to the systemic metabolic responses to amino acid‐modified diets at least in part.

**FIGURE 2:**
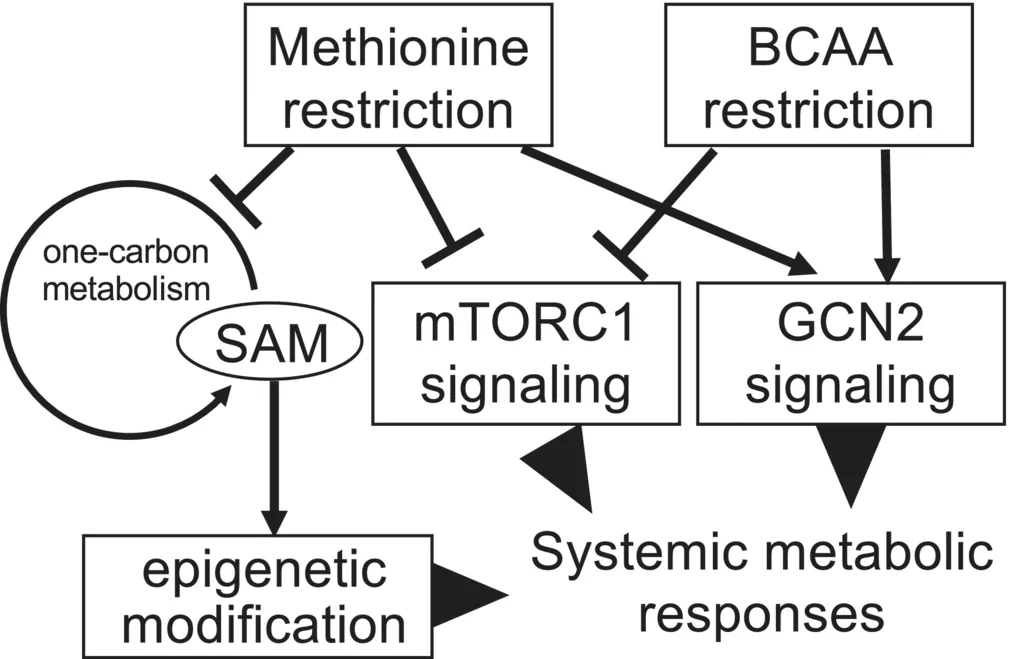
A brief overview of the effect of methionine and BCAA restriction. Methionine restriction perturbs the one‐carbon metabolism, causing SAM shortage, which modifies the epigenetic profile in cells. Methionine (or SAM) and BCAA (or leucine) are the established activators of mTORC1, which signals to the downstream effectors and causes diverse metabolic regulations. GCN2 kinase is also activated by methionine and BCAA restriction, which is also involved in the systemic metabolic responses. BCAA, branched‐chain amino acids; GCN2: general control nonderepressible 2; mTORC1, mechanistic target of rapamycin complex 1; SAM: S‐adenosylmethionine.

Arginine restriction (ArgR) and lysine restriction (LysR) are another notable dietary interventions. Both are categorized as alkaline (cationic) amino acids, and importantly, antagonistic properties between these amino acids have been reported in several species including prawn (X. J. Dong et al. 2018), some fish (Berge et al. 2002; Sun et al. 2025), chick (Austic and Scott 1975), rat (Ulman et al. 1981), and dog (Czarnecki et al. 1985), while not observed in young pigs (Edmonds and Baker 1987) and adult cats (Fascetti et al. 2004). The transport system of cationic amino acids on the cell membrane primarily involves cationic amino acid transporters (CATs) and heteromeric amino acid transporters (HATs) to a lesser extent (Closs et al. 2004, 2006). Arginine and lysine, as well as ornithine, are recognized and transported by these transporters with almost comparable affinity. Because those amino acids pass through the same machinery, cellular uptake or intestinal uptake of them occurs in a mutually competitive manner (Berge et al. 1999; Devé et al. 1998; Hosokawa et al. 1997). For this reason, relative excess of one of them in a diet may cause a restriction of the other, and thus, a balanced intake of the two amino acids is important. It is worth attention that ArgR, LysR, or unbalancing these amino acids is usually associated with the alteration of lipid metabolism, causing dramatic changes in lipid content in the liver, muscles, and blood stream in largemouth basses (Sun et al. 2025), turkeys (Ognik et al. 2020), rabbits (Giroux et al. 1999), rats (Goda et al. 2021; Nishi et al. 2018, 2020), and pigs (Katsumata 2011). In many cases, ArgR and LysR show milder or slight influence on body weight gain compared with MetR and BCAAR (Said et al. 1974), enabling relatively easier practical application to meat production.

Arginine and lysine are directly and indirectly involved in many metabolic processes in animals including urea cycle, tricarboxylic acid cycle, ketone body metabolism, and nucleobase metabolism. Because arginine is a precursor of ornithine, citrulline, nitric oxide, polyamines, proline, and creatine, and lysine is a precursor of carnitine, a number of research has been published attributing the ArgR‐ or LysR‐induced metabolic alterations to changes in those derivative metabolites (Wu 2009). Meanwhile, arginine is known as an mTORC1 agonist (X. Hu and Guo 2021). In addition, arginine can potentially bind to some transcription factors causing a conformational change, ending up with modulation of downstream gene expression (Geiger et al. 2016). Furthermore, amino acids including arginine are utilized in cells as a common posttranslational protein modification referred to as protein aminoacylation or arginylation, which strikingly modulates the target biological function and enables the cells to respond to the change in amino acid availability (He et al. 2017; Lin et al. 2025; Y. C. Yu et al. 2021). These results clearly indicate that not only do amino acid‐derived metabolites mediate the bioactivity in a canonical way but also amino acids per se participate in the metabolic regulation serving as signaling molecules.

### 3.2 Amino Acid Supplemented Diet

Although a restriction of some amino acids can be beneficial in certain circumstances to meat producers, removing a specific (micro)nutrient out of the conventional feed is almost impossible or very costly, and a holidic diet is much more expensive. This may keep farmers distant from adopting those methods. In contrast, adding a certain nutrient to the feed can often be relatively affordable, and thus easier to practice. As well as restriction, substantial amounts of evidence have shown that amino acid supplementation also exerts a variety of nutritional bioactivities. Amino acids that are the most extensively studied in this respect are again methionine, BCAAs, and arginine. Supplementing these amino acids in the feed can expectedly promote growth; increase milk production; enhance a secretion of some hormones such as GH, IGF‐I, and insulin; modulate redox state; and improve reproductive performance in many livestock species (Hodkovicova et al. 2023; Wu 2009). In addition, threonine supplementation is well documented regarding the maintenance of gastrointestinal health. Because intestinal mucin protein comprises large amount of threonine, orally ingested threonine is mostly absorbed and utilized in the intestine and its adequate intake is essential for maintaining intestinal mucosal integrity and barrier function (Faure et al. 2005; Law et al. 2007; Mao et al. 2011). Given that animal feeds made from plant‐based protein sources often contain less threonine and it is a major limiting amino acid in many livestock species, threonine supplementation is preferable in perspective of gastrointestinal performance as well as promotion of growth and reproduction.

Not as widely established, a variety of reports have indicated the notable effects of the other amino acids. For instance, dietary supplementation of tryptophan (Fernstrom 2016), tyrosine (Jongkees et al. 2015), and histidine (Thalacker‐Mercer and Gheller 2020) is often associated with neurological context and cognitive function, simply because they are precursors of important neurotransmitters. Supplementations of glutamine/glutamate (Luise et al. 2022; Watford 2015), glycine (Razak et al. 2017), and serine (Jiang et al. 2021) are implicated in one‐carbon metabolism, nucleotide metabolism, redox homeostasis, epigenetics, and so on; however, these observations are not more than descriptive, and therefore, the direct causality of intake of those amino acids at the molecular level has been still unclear. From the animal scientific point of view, the fact that amino acid supplements can affect amino acid metabolism and thereby modify the metabolite composition in meat may be worth attention. For example, each of amino acids and its derivatives has different unique tastes and flavors, as discussed below, and a slight difference in their composition makes considerable changes to complex savor, which is significant for meat quality and palatability (C. J. Zhao et al. 2016). Furthermore, as modulation of dietary proteins/amino acids dramatically affects sugar and lipid metabolism as well as amino acid metabolism, it can also contribute to complex aroma production through the Maillard reactions in meat (Sohail et al. 2022). For another example, many amino acids have been indicated to be involved in redox homeostasis, where glutathione plays a central role and its level dramatically changes in response to some amino acid intake (Gould and Pazdro 2019). Lipid oxidation in meat and meat products is a significant factor for their quality, and endogenous antioxidant property will partially determine their shelf‐life (Domínguez et al. 2019). Additionally, glutathione itself participates in the Maillard reaction, contributing to the production of meaty flavor compounds (Du et al. 2025). Collectively, dietary amino acid supplementation can have large influence not only on animal growth, reproduction, and milk production but also on the quality of meat and meat products.

Majority of knowledge about the impact of dietary amino acids has been advanced based on the studies using nonruminant species, because ruminants can live on protein‐scarce forages and biological demand for amino acids can be satisfied by rumen bacterial synthesis. Furthermore, rumen bacteria actively catabolize orally taken substances including proteins and amino acids and recycles into other bioactive compounds; thus, it is technically difficult to interpret the direct effect of dietary amino acids on the host metabolism. For this reason, the exact protein/amino acid requirement of ruminants still remains vague to date. Contrastingly, it is known that domesticated cows require ~12%–20% crude protein in their feeds, and the amino acid balance should be considered to maximize growth and yields, presumably because it is utilized to fuel optimal ruminal fermentation (Schwab and Broderick 2017). In addition, emerging evidences have suggested that feeding rumen‐protected amino acids, which are designed chemically or physically to resist ruminal microbial metabolism and to release amino acids in the intestine, possibly improves growth performance, feed conversion, and nitrogen balance in cows and lambs (Arriola Apelo et al. 2014; Liu et al. 2021; Luo et al. 2025). Although the efficacy of rumen‐protected amino acids has been still controversial and their application is at the laboratory level so far, amino acid supplements may help animal care and meat production also in ruminant animals, which warrants future research.

### 3.3 Complications in Amino Acid Nutrition

As discussed here, restriction or supplement of a certain amino acid in a diet exerts diverse influence on animal metabolism, many of which can potentially be helpful for efficient farming and quality control of livestock products in some occasion. However, most of those information lacks vital point of view; does modifications of a single amino acid always produce the same outcome in every circumstance? Evidences suggest that the answer is no. D. Raubenheimer and SJ. Simpson extensively discuss this point in their elegant review (Raubenheimer and Simpson 2016). They have pointed out that although the nutrition science made spectacular success in the early twentieth century in relation to the micronutrient‐related noncommunicable diseases, it has failed to combat the problems of rapid prevalence of obesity and associated cardiometabolic diseases today: They have also offered simple but very important perspective that “in emphasizing specific nutrients, it fails to take into account the fact that food components interact in complex ways to give rise to emergent properties of diets that are not explicable at the level of individual chemical parts.” This is also the case in the amino acid nutrition. Amino acids are the hub of many metabolic and biosynthetic pathways, and they metabolically closely interact, and several of them interconvert with each other. Not only in amino acid metabolism do amino acids participate, but also they are involved in virtually every biological system directly or indirectly including carbohydrate metabolism, lipid metabolism, vitamin metabolism, nucleic acid metabolism, redox metabolism, and so on. These facts apparently suggest that nutritional interaction in a diet between amino acids and the other dietary components should be considered carefully.

Specifically, the results of modulation of the same single amino acid in a diet reported from different research groups sometimes contradict with each other: MetR significantly ameliorates high‐fat diet‐induced fatty liver and dyslipidemia, whereas MetR plus high‐fat load in combination with choline‐deficiency is an established severe MAFLD model (Matsumoto et al. 2013; L. Wang et al. 2020); some studies show that BCAAs intake including leucine positively correlates with incidence of insulin resistance and type 2 diabetes under the Western diet regimen, whereas others demonstrate that dietary leucine supplement improves insulin sensitivity and glycemia under high‐fat regimen (Cummings et al. 2018; Y. Zhang et al. 2007; Zheng et al. 2016); ArgR significantly induces hepatic steatosis in normal fat regimen, whereas ArgR totally inhibits hepatic steatosis in high‐fat regimen (Nishi et al. 2024; Otani et al. 2020). These observations clearly illustrate that the metabolic effects displayed by a single amino acid modification in a diet strikingly depend on the background components of the diet. Furthermore, close interrelationship among amino acids and the other nutrients, both within and between tissues/organs, results in incredibly complicated metabolic responses (Hui et al. 2020; Jang et al. 2019): For instance, threonine supplementation leads to a marked elevation of plasma threonine levels, accompanied by a significant reduction of lysine, phenylalanine, and methionine levels (Boehm et al. 1998); ArgR leads to a significant upregulation of serum methionine and histidine levels, and downregulation of tyrosine levels, with arginine levels unaffected (Otani et al. 2020). These observations suggest that not only do dietary amino acids per se enter the body ending up with metabolic regulation but also the subsequently upregulated or downregulated metabolites can function.

Factors other than dietary components also have unignorable influence on the metabolic responses to dietary amino acids. Basically, a functional diet will not function sufficiently until it is ingested, and thus, appetite plays a determinant role. Because it is well known that amino acids themselves affect the central nervous system, being the primary effector of appetite, satiety, and palatability (Fromentin et al. 2012; Morrison and Laeger 2015), dietary amino acid modulation to achieve target phenotype of animals may simultaneously affect food intake as a side effect, which can possibly ruin the desired outcome. Furthermore, although most proteins have little flavor and savor, free amino acids present a variety of taste (e.g., sweetness, bitterness, saltiness, and umami), which also evokes preference/aversion response in animals (Bachmanov et al. 2016; Harmon et al. 2024; Kawai et al. 2012; Nelson et al. 2002; G. Q. Zhao et al. 2003). In general, animals recognize bitterness through the large family of bitter taste receptors (T2Rs) and sweetness and umami through the sugar/amino acid taste receptors (T1R1, T1R2, and T1R3) (Vijayashree and Sivapathasundharam 2026; G. Q. Zhao et al. 2003). The repertoire of functional T2Rs differs substantially among vertebrates, and because each receptor responds to a single tastant with different sensitivities like a gradient, animals are able to perceive complex bitter tastes (Antinucci and Risso 2017; D. Dong et al. 2009; Harmon et al. 2024). In addition, a few mutations or small variations in peptide sequence of T1Rs result in a considerably different response to amino acids and sweeteners, suggesting that animals having a different genetic background will not necessarily display the same reaction to a certain tastant (Bachmanov et al. 2016; Nelson et al. 2002). In line with this notion, taste perception is actually diverse among animal species, and evidence from comparative biology suggest that it has evolved in a lineage‐specific manner, adapting the species to their habitat and feeding ecology (Antinucci and Risso 2017; Breslin 2013; Vijayashree and Sivapathasundharam 2026). For instance, obligate carnivores (e.g., cats) often lack sweet taste perception, presumably because they selectively eat meat containing high levels of proteins and amino acids and low in simple sugars; birds are often indifferent to sweetness, supposedly because they swallow foods whole and they do not have much need for tasting; omnivores and herbivores are generally more sensitive/diverse in bitterness perception than carnivores, likely to cope with a wider variety of food they consume (Antinucci and Risso 2017; Vijayashree and Sivapathasundharam 2026). These observations suggest that livestock species including many taxa are likely to exhibit species‐ and strain‐dependent preference to nutrient‐modified feed, and thus, careful attention should be paid to keep or maximize feed intake. Moreover, environmental factor such as ambient temperature also affects food intake considerably. Cyclic heat stress with high temperature and humidity in the daytime (e.g., during summer season in Asian countries) will markedly decrease the meal size of animals (de Oliveira et al. 2023). Further, metabolic effects of protein/amino acid ingestion can vary depending on feeding pattern (ad libitum vs. time‐restricted) and meal distribution (bolus vs. even, or breakfast vs. dinner) (Aoyama et al. 2021; Areta et al. 2013; Negri et al. 2025).

For the practical application of protein/amino acid and to maximize the efficacy, all these factors should be taken into account; yet, there are too many to handle. In such problems, rapidly progressing mathematical modeling and artificial intelligence (AI) technologies strongly help us in decision‐making on hundreds of variable parameters. Experimental technologies for quantifying biological responses and computational methods for extracting relationships from high‐dimensional data have developed in a mutually reinforcing manner in the last decade. These new technologies have already made a great contribution in some sectors of agriculture (Distante et al. 2025; Menezes et al. 2024). In the next chapter, recent advances in measurement/analytical methodologies and their applications in animal science and amino acid nutrition are reviewed.

## 4 Systems Biology Perspectives on Animal Amino Acid Nutrition

Recent advances in omics technologies have enabled comprehensive profiling of genes (transcriptome), proteins (proteome), and metabolites (metabolome). Systems biology is a discipline that aims to understand, model, and predict biological functions by integrating comprehensive measurements of molecular and physiological components (Kitano 2002; Nielsen 2017). These technologies and conceptual frameworks provide valuable insights not only for fundamental biology but also for applied fields such as livestock management and precision agriculture (Abd El‐Hack et al. 2025; Ashokan et al. 2023; H. Hu et al. 2021; Wadood et al. 2025). For instance, metabolomic data for major livestock species (e.g., cattle, sheep, goats, horses, and pigs) are publicly available through an open‐access livestock metabolome database (Goldansaz et al. 2017). Metabolomics is a key approach for simultaneously quantifying numerous metabolites, typically using mass spectrometry (MS) coupled with chromatographic separation techniques such as liquid chromatography (LC) and gas chromatography (GC), as well as nuclear magnetic resonance (NMR) spectroscopy (Roessner and Bowne 2009). Because metabolomic profiles closely reflect physiological states, metabolomics has been widely applied in animal science and livestock research (Abd El‐Hack et al. 2025; Goldansaz et al. 2017).

### 4.1 Metabolomics Analysis and Methodologies

Metabolomics enables biomarker discovery, sample classification, and the identification of biochemical pathways and metabolic networks. A biomarker is defined as “a biological molecule that serves as an indicator of a normal or abnormal process, state, or disease” (Green et al. 2023). In veterinary medicine and animal science, biomarkers reflecting the physiological and production status of livestock are highly valuable (Green et al. 2023). Metabolomics data are typically analyzed using multivariate methods such as principal component analysis (PCA) and partial least squares discriminant analysis (PLS‐DA) to detect differences between groups. These analytical methods allow the identification of factors, such as metabolites, that determine differences between groups from among numerous factors (Tao et al. 2023). Although univariate analyses are informative, single metabolites are often insufficient as robust biomarkers (Nagana Gowda and Raftery 2013). Therefore, multivariate statistical and machine learning models are increasingly used to improve biomarker prediction performance (Ahmad et al. 2024; Nagana Gowda and Raftery 2013; Woldeamanuel et al. 2024). Much of metabolomics research has focused on accurate sample classification and biomarker discovery. In contrast, pathway and network analyses focus on the relationships among metabolites (Amara et al. 2022).

Enrichment analysis maps metabolites onto predefined metabolic pathways to facilitate biological interpretation (Schermer et al. 2025). Network analysis is primarily divided into two types: knowledge‐based and experiment‐based (Amara et al. 2022). Knowledge‐based networks are constructed from curated biochemical knowledge, providing a framework for interpreting metabolomics data in light of existing biological information such as metabolic pathways and enzyme reactions. For example, metabolic networks, as exemplified by KEGG (Kyoto encyclopedia of genes and genomes) pathways (Kanehisa and Goto 2000), are a form of knowledge network. In metabolic networks, metabolites and biochemical reactions are represented as nodes and edges. Enzyme reactions closely link metabolic enzymes and metabolites, potentially making interpretation easier through database information such as EC numbers (McDonald and Tipton 2023) and Rhea IDs (Bansal et al. 2022). On the other hand, data‐driven (experimental) networks are inferred directly from metabolomics datasets based on correlations identified between metabolite quantities within the data (e.g., correlation networks). Experimental networks offer the advantage of enabling metabolite classification and feature extraction without requiring prior biological information. Various network types can be constructed depending on experimental design and statistical assumptions, and when combined with methods such as centrality analysis, they can also be applied to biomarker discovery (Carmelo et al. 2020; Fujita et al. 2022; Werner et al. 2019). When combined with appropriate experimental design and advanced statistical and graph‐based methods, metabolomics provides a powerful framework for investigating metabolic regulation while preserving inter‐metabolite relationships.

### 4.2 Biomarker Discovery for Amino Acid Nutrition

The metabolome of livestock, particularly blood amino acid profiles, exhibits significant changes when the dietary amino acid composition and quantity are altered. These metabolomic changes can be considerably associated with lipid metabolism, hepatic steatosis, and intramuscular fat deposition in various livestock species including cattle, pigs, sheep, and poultry (Abd El‐Hack et al. 2025; Connolly et al. 2019; Goldansaz et al. 2017; Guo et al. 2025). This section reviews associations between dietary composition, circulating metabolomes, and meat quality traits in several livestock species, serving as examples for biomarker discovery and precision agriculture research.

In animal production research, the relationship between feed intake and growth performance is a central topic (Taiwo et al. 2021; Xu et al. 2017; X. Zhang et al. 2022), and investigating the correlation between blood metabolites and meat quality as well (Connolly et al. 2019; Yamada 2022). Metabolomics data based on LC–MS technology have been widely used to identify blood metabolic signatures associated with residual feed intake in beef cattle (Taiwo et al. 2021). This study found that arginine biosynthesis and histidine metabolism pathways were enriched in highly feed‐efficient steers compared with least‐efficient animals (Taiwo et al. 2021). Five candidate biomarkers were identified: methionine, methionine sulfoxide, 5‐aminopentanoic acid, 2‐aminohexanedioic acid, and 4‐chlorolysine, all primarily associated with amino acid metabolism.

Studies on Wagyu cattle have reported that associations between blood metabolomic profiles and carcass characteristics have identified seven blood metabolites positively correlated with marbling: 3‐hydroxybutyrate, propionic acid, acetic acid, creatine, histidine, valine, and isoleucine. This study demonstrates that blood metabolomics aids in understanding fat and muscle metabolism in cattle (Connolly et al. 2019).

Systems biology approaches have also been applied in sheep production as a strategy to enhance productivity in milk, meat, and wool production (Ali et al. 2024; El‐Sabagh et al. 2016). Ali et al. demonstrated using sheep that high‐protein feed significantly alters the ruminal metabolite profile. Using PCA and PLS‐DA, they demonstrated that over 40 metabolites differed significantly between treatment groups, with organic acids being the most affected metabolites. Major amino acids such as aspartic acid, phenylethylamine, tyrosine, tryptophan, and methionine sulfoxide showed an increasing trend with rising feed protein levels (Ali et al. 2024).

### 4.3 Multi‐Omics Integration and Systems‐Level Analyses

Technologies for simultaneous measurement and integration of multiple omics layers, including host and microbial communities, are rapidly advancing in livestock research (Hasin et al. 2017; Subramanian et al. 2020; Woelders et al. 2011). For example, the metabolic state of cows producing high‐protein milk has been characterized using combined metabolomic and microbiome analyses (Xue et al. 2020). Integrating interaction networks derived from metabolic pathways with transcriptomic and genomic data enables a more comprehensive understanding of metabolic regulation (Baião et al. 2025). Other approaches infer regulatory relationships linking genomic and transcriptomic variation to metabolic phenotypes (Nishida et al. 2024). Such methodologies are increasingly applied to studies of meat quality traits in cattle, swine, and poultry as techniques for improving meat texture and flavor (Hwang et al. 2023).

Multi‐omics integration revealed site‐specific variations in intramuscular fat in Qinchuan cattle (H. Yu et al. 2023). This study performed an integrated analysis of muscle transcriptomes and metabolomes across four beef cuts having variable intramuscular fat levels sampled from the same individual, and using multivariate and network analyses, coordinated regulation of unsaturated fatty acid and amino acid metabolic pathways was identified. Similar studies have identified associations between metabolic phenotypes and underlying genetic variation (Verardo et al. 2023). These studies primarily rely on knowledge‐based network frameworks, depending on prior knowledge and database expertise, while facilitating physiological interpretation.

In an integrated study of chickens (Goto et al. 2021; J. Ma et al. 2025), researchers combined liver and abdominal fat transcriptomics with serum metabolomics to identify key regulators of abdominal fat deposition and they found several genes (e.g., FOXF1 and USP10) and metabolites (e.g., tyrosine) as the key factors. Correlation network analysis suggested a shift from lipid biosynthesis to fatty acid oxidation in the low‐fat strain, with medium‐/long‐chain fatty acids levels suppressed. Through the analyses, USP10 emerged as a potential core regulator of abdominal fat deposition and metabolic homeostasis.

Age‐dependent variations in amino acid metabolism in porcine liver and intestinal tissues have also been addressed through a multi‐omics approach (Guo et al. 2025; Liaubet et al. 2023; You et al. 2025). Fluctuating concentrations of 31 hepatic metabolites, which were involved in tryptophan and glutamate metabolism, were observed, and strong correlations were detected between amino acid metabolites and specific colonic microorganisms, which highlight the importance of interorgan communication (gut–liver) in amino acid metabolism. For large livestock animals, continual collection of blood, urine, saliva, feces, milk, and small pieces of tissue (biopsy) from the same individual is relatively easy; thus, it enables farmers in support of computing methods to monitor the temporal dynamics of animal metabolism and to grasp (or predict) the animal/meat condition at any stages. Although multi‐omics integration and network analysis provide mechanistic insights, predictive modeling using AI represents a complementary framework for translating omics data into actionable nutritional strategies.

### 4.4 AI Approaches for Omics‐Based Prediction

Majority of omics research has primarily focused on correlations among biological variables. However, in recent years, AI and machine learning approaches have increasingly been applied in systems biology to predict metabolic phenotypes from complex datasets (Randall et al. 2023; L. Zhu et al. 2025). This trend is also emerging in livestock research (Bao and Xie 2022; S. Zhang et al. 2025; X. Zhao et al. 2025). In pigs, machine learning models using metabolomic profiles from urine, saliva, and serum have been developed to predict reproductive performance in females (Fletcher et al. 2026). Feature selection using supervised machine learning algorithms such as support vector machines and random forests has been applied to identify key genes associated with intramuscular fat accumulation in pigs (Shi et al. 2024). A support vector machine is the one which identifies variables that separate different sample groups most effectively, and a random forest is the one which ranks variables according to their contribution to prediction accuracy by integrating many independent predictive models. We previously developed a neural network model to predict hepatic triglyceride levels from serum amino acid profiles in rats fed diets with different amino acid compositions (Nishi et al. 2018). Machine learning studies integrating porcine transcriptomic data with fat‐related phenotypes have applied random forest and gradient boosting models to identify predictive biomarkers (Liu et al. 2022). These models identified biomarkers, including amino acids and lipid intermediates, predictive of fat content and meat quality traits.

Collectively, these studies demonstrate the potential of AI‐based models to predict metabolic responses to specific stimuli, including dietary amino acid manipulation. Therefore, integrating comprehensive dietary data with physiological and omics parameters, combined with multivariate and machine learning models, may enable prediction and optimization of metabolic responses. To advance this field, it is essential to foster interdisciplinary research teams with expertise in both experimental animal science and computational data analysis.

## 5 Future Perspectives

Historically, most findings in nutrition science have aimed to understand humans with particular focus on rodents, primates, and other small mammals like dogs as “model” organisms. However, as comprehensively discussed herein, growing evidence have underpinned the translational relevance of that information to livestock species. Because the essence of metabolic responses to dietary amino acids is conserved and shared in common among major animal species including fish, poultry, pig, and presumably ruminants, precise control over dietary amino acid composition offers the potential to enhance feed‐to‐gain efficiency, optimize carcass composition, and regulate meat characteristics such as flavor, marbling, and texture. Traditionally, high‐protein feed formulations have been preferred in livestock industry to maximize yield and cost effectiveness; however, given the latest knowledge on animal nutrition, just adding proteins is not the only way to gain the equivalent consequence, and sometimes even reducing protein or amino acids to a certain extent may be preferable. In agriculture, vast amounts of nitrogen‐source are put in every day and unused residual nitrogen emitted to the environment will ultimately pollute soil, water, and atmosphere (Siddiqui et al. 2023). Therefore, strategic dietary amino acid manipulation can help improve nitrogen efficiency and reduce the need for burdensome protein‐rich feed ingredients, thereby contributing to more sustainable livestock production and food systems (Leip et al. 2021). The ability to control the meat quality as one wishes by manipulating amino acid balance in animal feed offers a powerful tool for precision agriculture; it enables producers to make value‐added products, enhance product differentiation, and respond to evolving market demands for ethically and sustainably produced animal products. Integration with food technology and metabolomics platforms may further enable real‐time monitoring of animal metabolic states, facilitating adaptive feeding strategies that balance production efficiency with product quality, which ideally leads to custom‐made livestock production.

Although accumulating evidence supports the conserved role of amino acid sensing and corresponding metabolic regulation across species, direct translation from model organisms to livestock animals still remains challenging today, because final phenotypic outcomes are often modified by species‐specific physiology, developmental stage, production purpose, environmental conditions, and so on. We believe that the systems biology approach provides a promising framework to bridge this practical gap. By integrating multi‐omics datasets, metabolic network analysis, physiological measurements, and machine learning techniques applied across species, the core conserved regulatory modules governing amino acid metabolism can confidently be distinguished from species‐specific adaptations. Such integrative models would enable to predict the metabolic responses in livestock animals based on the minimum of experimental data and established mechanistic knowledge of rodents and other model organisms, thereby helping reduce substantially the need for extensive feeding trials. Ultimately or ideally, this systems‐level translation would accelerate the rational design of precision nutritional strategies tailored to each livestock species. The biggest limitation to date is the cost of adding/omitting amino acids and toughness of handling huge data and its server; thus, cooperative development in basic science, food technology, and agricultural sector are vital to accomplish future food innovation.

## Author Contributions

**Hiroki Nishi**, **Suguru Fujita**, and **Fumihiko Hakuno** wrote the manuscript. All authors have read and agreed to the published version of the manuscript.

## Funding

This study was supported by the Japan Society for the Promotion of Science (JSPS) KAKENHI, 23K13923 granted to H.N., 26K18010 granted to S.F., 15H04583, 23H00358, 26K23093, and 23K23793 granted to F.H.

## Conflicts of Interest

The authors declare no conflicts of interest.

## Data Availability

Data sharing not applicable to this article as no datasets were generated or analysed during the current study.
